# How Might We Best Spend the Winter Sessions of Our Association?

**Published:** 1894-11

**Authors:** 


### HOW MIGHT WE BEST SPEND THE WINTER SESSIONS OF OUR ASSOCIATION?

In such a topic I have found rather a suggestive one, and apropos of the time and place, particularly when we seem drifting around uncertain as to what is best to do, and consider, and so many prominent questions knocking at our doors for solution.

While our work last year was pleasant, our meetings enjoyable, our social feeling strengthened, still, as perhaps one of the oldest local societies in our country, the sum total of our work was but a little offering from such a body, and I hope it will be the least of any year in the history of our growth.

With the ever-changing needs and growth of our profession, its ever-increasing usefulness, and the extended fields of labor, with the rich harvests to be garnered, we should be active, vigilant and earnest in the part that we should play, and our voice should be sounded aloud in the forward progress of these movements that mean so much for the welfare of our people and the successful growth of our great country.

Let us ask ourselves how much we are doing; how much we should do; how well we are sustaining and encouraging a higher intellectual growth of our profession for the future. Are we not, as organized bodies, largely responsible for the condition and status of the veterinary colleges of our land? Does it not seem like a waste of energy to rail at the output of our schools, when by no direct act of ours have we made any distinction between these seats of learning; when we have failed, save on perhaps a single occasion, to recognize the advanced steps taken by old schools; the better promised curriculum of new schools. Should we not, annually, devote a part at least of one meeting to consideration of the American veterinary colleges, and thus be an element of strength in bettering American veterinary education?

With our advanced schools of three years, of six to eight months each, giving an education to-day superior to many of the foreign schools, is it not our duty to give stronger recognition to this work, and thus give at home a better conception of the work of our colleges, a higher appreciation of our diploma, that we may early look forward to its proper recognition abroad? Are we not unmindful of a condition, often aggravating in the extreme? Our own nation open for reception of veterinary graduates of every foreign school; our laws actually formulated to place greater freedom, higher value in their possession than those of our own. Yet should one of us enter a foreign country with our sheepskin we would hardly get farther than a port of entry. All American veterinary schools are, *in their* conception, on the same level, and are we not largely responsible for this lack of knowledge on their part? A careful and proper discrimination, on our part, would soon make a different state of affairs, and our deserving colleges would soon meet that just reward of recognition to which they are so justly entitled. Many other points of equal value and importance might well be considered by us, from time to time, in the meetings of this Association, but I will not take time to discuss them now.

Many are the complaints of the rapidly multiplying forces in our calling, and much severe criticism is heard now and again. Why waste our energy in this direction, when perhaps 90 per cent. of these new recruits are better fitted to practice, by virtue of better educational facilities than we have ever had? Why not rather try to encourage the employment of skill by every horse owner, and drive out the ignorant pretenders and experimenters who disgrace our calling and poison the minds of horse owners against the employment of any true veterinary service? Again, why should we complain at this, for it must be, in many instances, by better work that they encroach on our territory of labor? The survival of the fittest; supply and demand regulate after all the markets and field of labor the world over Let us agitate for a wider sphere of employment for the veterinarians, and soon we will find the field so large that there will not be minds and hands sufficient for the work. The flesh and fluids used by man for the sustenance of life carry with them the greatest dangers to its existence and well being, and to no one occupation does the great field of this work so naturally fall as to the veterinarian.

We speak of living in an age of progress, an era of inventive genius, but what of all this if we are daily denied a full enjoyment of these blessings by impaired vigor and health? What a sad defect in our intellectual advancement; what a comment upon our boasted learning; when there may be paraded on our stalls the most unwholesome flesh for our consumption; when there may be peddled through our streets, lanes and roads, milk from diseased cattle, produced under the most unsanitary conditions and surroundings, and all of this in a land where there is no limit to the production of these life-giving elements at the cheapest cost on earth. Why, the very fowls upon our table, the toothsome squab, may all be the natural carriers, or vehicle of transmission of disease and death, and what is everybody's business is nobody's, and we bemoaning the increase of workers, and scarcely a veterinary sanitary police in the State. Let us be up and active in agitation for laws and ordinances to regulate these matters with some thought and interest for the public welfare, and soon we will find the measure of our worth weighed in a different balance. Let us no longer be lulled into a false sense of security, our vision obscured by such fatuitous reasoning that our veterinary sanitary service has cost yearly but a few thousand dollars, and death and destruction goes on among our families, our children, and those dear to us by ties of friendship. We are terrorized over the possibilities of Asiatic cholera; made to tremble over the probabilities of small-pox; yet dulled to a sense of indifference to an almost criminal point, because it was ever thus, as one out of every eight deaths in our State are victims of tuberculosis, not counting those hundreds of little lives lost, enumerated under the head of inanition, marasmus, cholera infantum, tabes mesenterica, and the many allied forms of disease, which have their origin in an unwholesome milk supply and an utter lack of surveillance of these centres of production. Have we not had enough examples of outbreaks of typhoid fever, diphtheria, scarlet fever, and many other less serious because less fatal, to wait any longer for better sanitary inspection of such fields of danger? Have we not suffered, as a people, long enough from these dangers? Have we not lessened the energy, curtailed the abilities, destroyed enough of the lives of our people, to warrant more aggressive legislation in this direction? Are our minds so narrowed and warped that we are silenced by a criticism that proclaims that the State is to be saddled with an annual expense of fifty thousand dollars; that would not measure the loss of the lives, each one of you know personally, whose origin, perpetuation, early fatality, was in whole, or in part, due to an unwholesome meat and milk supply of our commonwealth? We have too long searched for reasons and excuses why we should not do this or that; too long listened to the opinions of those who wish to cloud in obscurity these public movements, and finding some little doubtful point, backed only by conjecture, rise up as an objector and stay every movement in this direction for the public good. Are the selfish interests of a few people, who care little for the public health, so their own selfish advancement is not curtailed, to control us? Let us no longer wait for the settlement of details and modes of procedure, but make a firmer and stronger demand for laws recognizing these existing conditions and legislative enactments for their restraint and control through properly created bodies.

I have heard some complain because they had so little to do; yet they were too busy to be better preparing themselves for what they might have to do. Our journal columns are not full to overflowing, yet they are all worthy of our support and should be read by every one. The preparation of concise records of our cases will do us all much good, be a source of instruction, and better fit us for that work we have all pledged ourselves, as members, to help one another by the dissemination of knowledge. For our association we could each find some one case monthly to interest each other, and also add contributions to our journalistic columns that would tend to strengthen our profession and make our association stronger in interest to every member.

My sanitarium is always at your command for the reception of any cases of peculiar interest, that might be brought here for clinical use on the evening of our meetings. Thus we might use this means of entertaining and instructing each other, so that our coming together would help and aid in our daily work.

We have long had a question box, but it is unused, and I am sure not a week passes over the head of any one of us but what we would like to have the opinion of some other fellow veterinarian upon some obscure case or doubtful point, and this is a means to this end. Why leave it again unused?

Another line of work that we might well take up, and that would be of much interest, would be, say, the experience of members in dealing with some one disease. For instance, thirty minutes might be devoted of each meeting this winter in hearing reports from each member, of the cases of azoturea he has dealt with the preceding thirty days, the number of semi-paraplegic cases, the number of entire paraplegia, those involving the forelimbs, together with the treatment of each case, and the results obtained. This plan might be applied to such diseases as cerebrospinal meningitis, puerpera hemorrhagica, tetanus, and many others of equal interest and importance.

We have among our members a number who are doing sanitary police work, and interesting reports and descriptions of their work might be brought before us from time to time.

---

The Civil Service Commission will hold an examination on Friday, December 21st, in Washington and other large cities, for the position of Meat Inspector, at $1,400, and Assistant Inspector, at $1,200, in the Agricultural Department. Only graduates of veterinary colleges will be admitted.

